# Taxonomic Distribution of FosB in Human-Microbiota and Activity Comparison of Fosfomycin Resistance

**DOI:** 10.3389/fmicb.2019.00200

**Published:** 2019-02-13

**Authors:** Ziwei Song, Xue Wang, Xingchen Zhou, Su Jiang, Yuanyuan Li, Owais Ahmad, Lianwen Qi, Ping Li, Jing Li

**Affiliations:** ^1^School of Life Science and Technology, China Pharmaceutical University, Nanjing, China; ^2^State Key Laboratory of Natural Medicines, China Pharmaceutical University, Nanjing, China; ^3^School of Traditional Chinese Pharmacy, China Pharmaceutical University, Nanjing, China; ^4^Key Laboratory of Drug Quality Control and Pharmacovigilance, China Pharmaceutical University, Nanjing, China; ^5^Clinical Metabolomics Center, China Pharmaceutical University, Nanjing, China

**Keywords:** FosB, fosfomycin, antibiotic resistance proteins, gut microbiota, *Staphylococcus*

## Abstract

FosB, a Mg^2+^ dependent thioltransferase, confers antibiotic resistance to fosfomycin through enzymatic drug inactivation. Among all antibiotic resistant proteins in the Antibiotic Resistance Genes Database and the Comprehensive Antibiotic Resistance Database, FosB is within 5% of the most number of ARPs identified in Human Microbiome Project reference database but mainly distributed in limited genera, i.e., 122 of total 133 FosB homologues are found from *Bacillus* and *Staphylococcus*. Furthermore, these FosB sequences could be divided into three clusters based on their phylogenetic relationship, i.e., two groups of FosB were mainly from *Bacillus*, and another was mainly from *Staphylococcus*. Finally, we confirmed that FosB from the group of *Staphylococcus* presented the highest resistance ability to fosfomycin by *in silico* and *in vitro* comparisons. In summary, this study elaborates the specific taxonomic characteristics and resistant abilities of FosB in human microbiota, which might help in developing more promising fosfomycin-like antibiotics.

## Introduction

Antibiotics are crucial measures in the treatment of infectious diseases. However, the irrational use of drugs and long-term abuse of antibiotics can lead to bacterial resistance. The number of infections caused by multidrug-resistant bacteria has increased during the last decades, and it has been presenting a potential threat to human health (Norrby et al., 2005; Cosgrove, 2006). Therefore, research on multidrug-resistant bacteria has drawn more and more attention globally.

Known mechanisms of bacterial resistance to drugs in the human body can be summarized into six major categories: production of inactivated enzymes and modified enzymes (Zhang et al., 2012; D’Andrea et al., 2013), changes in the target site of the drug (Leclercq and Courvalin, 2002), formation of the outer membrane’s permeability barrier (BorgesWalmsley and Walmsley, 2001), activation efflux of antibiotics pump (Sobel et al., 2005), formation of the bacterial biological membrane (Mah and O’Toole, 2001), and cross-resistance (Via et al., 2010), respectively. Typically, the bacterial enzyme which can inactivate antimicrobials is the most important and well-studied mechanism of drug resistance (Michalopoulos et al., 2011). Meanwhile, the focus of many studies has been on the bacterial resistance to aminoglycoside and β-lactam antibiotic (Michalopoulos et al., 2011). However, as an ideal broad-spectrum antibacterial drug that has been clinically available for decades, there are only a few studies of fosfomycin on the distribution of drug-resistant genes.

Fosfomycin resistance is caused by plasmid-mediated fosfomycin genes in many bacterial species. Currently, four main types of fosfomycin-resistant genes are known. They are *fosA* (Beharry and Palzkill, 2005)*, fosB* (Fu et al., 2016)*, fosC* (García et al., 1995) *and fosX* (Fillgrove et al., 2007). FosB, the Mg^2+^ dependent thioltransferase encoded by *fosB*, can form a complex by catalyzing the nucleophilic addition of L-cysteine or bacillithiol to C-1 of fosfomycin, leading to the fosfomycin resistance (Roberts et al., 2013).

In previous studies, *fosB* were in Gram-positive organisms such as *Staphylococcus* *aureus* and *Bacillus* *subtilis* (Cao et al., 2001; Thompson et al., 2014). Recently, some researchers have published about the influence of *fosB* in the vancomycin-resistant *enterococci* strains (Sun et al., 2017). However, the distribution and characteristics of FosB sequences in human microbiota are still unclear. It might be complicated and time-consuming to screen *fosB*-containing bacteria systematically through traditional experimental methods.

The taxonomic distribution of FosB can be explored comprehensively by using the available high-throughput sequencing technology and the public genomic database. For example, the Antibiotic Resistance Genes Database (ARDB) includes most of the publicly available antibiotic resistance proteins (ARPs), which can provide reliable annotations for researchers to investigate the molecular mechanism of bacterial resistance (Liu and Pop, 2009). The Comprehensive Antibiotic Resistance Database (CARD) contains all the ARPs’ information in the ARDB and is updated monthly to ensure data validity (Mcarthur et al., 2013). On the other hand, the Human Microbiome Project (HMP) provides the baselines and variances of human gut metagenomic data, which is aimed at ensuring the comprehensive characterizations of the human microbiome (Proctor, 2016). Thus, the combination of public databases with sequencing technology will help us to investigate the mechanisms of resistant genes in detail.

In our study, we mainly focused on the unique ARP by comparing the number of homologous genes and taxonomic diversity of ARPs in ARDB and CARD, i.e., FosB exhibited more homologues among all ARPs but was distributed in limited bacteria. The taxonomic distributions of all FosB in human microbiome were investigated systematically, and the representative sequences were selected to compare the structures and fosfomycin resistance. These findings are crucial not only to understand the fundamental principles of bacterial resistance but also to provide more effective strategies to control the proliferation of resistant organisms and even eliminate their resistance.

## Materials and Methods

### Access to ARPs

Calculated sequences of ARPs were taken from ARDB and CARD. The ARPs from ARDB were clustered approximately into 380 categories according to the distinct gene types. MetaGeneMark (v2.8) predicts the genes and related protein sequences of these bacterial genomes. The HMP reference database, https://www.hmpdacc.org/, contained 1,751 bacterial strains of 1,253 species in September 2014. Basic local alignment search tool (BLASTP) (Camacho et al., 2009) was used to conduct sequence alignment of FosB homologues with HMP database with a parameter of *e*-value = 1e-05.

### Phylogenetic Tree

Maximum likelihood (ML) method in MEGA software (v7.0) was employed in building the phylogenetic tree. It was further trimmed and edited by Dendroscope (v3.4.7).

### Multiple Sequences Alignment and Secondary Structures Prediction

The alignment among three representative protein sequences was conducted using DNAMAN (v8.0), and colors were modified as needed. The prediction of secondary structures of these proteins were completed by the PSIPRED Protein Sequence Analysis Workbench, http://bioinf.cs.ucl.ac.uk/psipred/.

### Computational Binding Analysis

The 14 known 3D structures of FosB homologues and their protein sequences were collected from Protein Data Bank database (PDB), https://www.rcsb.org/. The homology modeling was performed using the MOE (v2014.0901). And the molecular docking was completed using Autodock (v4.2.6) to generate 100 docked conformations for each ligand bound to its target (Morris et al., 2009). For each complex, the top 5 ranked docking poses were optimized in the binding pocket and used as the initial geometry in the molecular dynamics (MD). We performed 10 ns MD simulations to obtain the lowest free energy of binding for each complex, respectively. All the MD simulations were carried out with AMBER (v14.0) (Case et al., 2005). The final complex structures were demonstrated by PyMOL (v2.1.1).

### Construction and Expression of *fosB*-Recombinant Strains

For the expression of these three proteins of FosB, the representative *fosB* genes were cloned into pET28a (+) vector by NcoI/XhoI-digested, respectively. Then the recombinant plasmids were transformed into *E. coli* BL21 (DE3) competent cells. The recombinant strains were named as FosB*-*b1, FosB*-*b2, and FosB*-*s, respectively.

The three *fosB*-recombinant bacteria were inoculated into Luria-Bertani (LB) broth containing 50 μg⋅mL^-1^ kanamycin and incubated at 37°C. The FosB expression of recombinant was induced by isopropyl β-D-thiogalactopyranoside (IPTG, 0.5 mM) and then the inoculums were collected for detection of resistant abilities.

### Kirby-Bauer Susceptibility Testing

Kirby-Bauer susceptibility testing was performed based on the Clinical Laboratory Standards Institute (CLSI) (Hudzicki, 2009). *E. coli* BL21 (DE3) was used as control strain. The turbidity of all bacterial suspension was adjusted to a bacterial concentration of 0.5 McPherson (about 10^8^ cfu⋅mL^-1^) by LB broth. A sterile cotton swab was streaked onto the Mueller-Hinton (MH) agar plate and the entire agar surface was drawn. The fosfomycin susceptibility test paper (200 μg fosfomycin per paper, with 50 μg glucose-6-phosphate) was applied to the surface of the plate. The diameter of the inhibition zone was measured with vernier caliper after 18 h of incubation at 35°C.

### Detection of Resistant Ability

The resistance to fosfomycin was determined by measuring optical density at 600 nm (OD_600_). The reaction system contained 20 μL fosfomycin solutions and 180 μL freshly grown bacteria. The concentration of recombinant bacteria was 10^6^ colony-forming units (cfu)⋅mL^-1^ (Bär et al., 2009). Fosfomycin solutions with a final dilution range of 0, 16, 32, 48, 64, 80, 96, 112, 128, 144, and 160 μg⋅mL^-1^ were used, respectively. The OD_600_ values of three *fosB*-recombinant strains and *E. coli* BL21 with different concentrations of fosfomycin were detected per 2 h over 12 h to determine minimum inhibitory concentrations (MICs). 20 μL of the above cultured solution was used to smear the LB petri dish, and the minimum bactericidal concentrations (MBCs) of *fosB*-recombinant bacteria were determined by observing colonies growth after culturing at 37°C for 12 h.

## Results

### The Unique Taxonomic Diversity of FosB Among All ARPs

**FIGURE 1:**
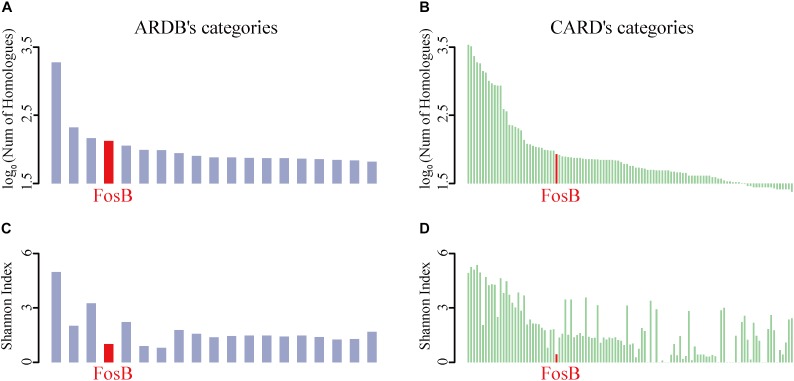
The taxonomic number and diversity of FosB. **(A)** The top 5% ARPs’ categories of log-transformed number of homologues in HMP reference database among ARDB and **(B)** CARD, respectively. **(C)** Shannon index of top 5% ARPs’ categories among ARDB and **(D)** CARD, respectively. Light purple and light green column represent the log-transformed number of homologues in HMP reference database and Shannon index of ARPs among ARDB and CARD, respectively. Red highlighted the information of FosB.

To identify the distribution of all ARPs, the number of homological sequences of ARPs identified from the HMP reference database was calculated firstly (Figure 1A,B). It was shown that BacA, AcrB, TetC, FosB, and KsgA were the top 5% categories with the most number of homological sequences from ARDB (Supplementary Table S1). Meanwhile, FosB was also one of the ARPs with a lot of homologous sequences from CARD, which contained 85 homologues (Supplementary Table S2).

The Shannon Index of ARPs could reflect the diversity of taxonomic distributions of them in ARDB and CARD, i.e., the lower Shannon Index of ARPs indicated the limited distribution of species (Figure 1C,D). Interestingly, the results from the two databases showed that FosB was the only ARP that showed more homologues while distributed in fewer bacterial species in human microbiota.

### The Taxonomic Distributions of FosB in Human Metagenomes

133 FosB homologues were identified from the HMP reference database based on the known FosB sequences from ARDB, while only 85 FosB homologues were found in CARD entirely (Supplementary Table S3).

**FIGURE 2:**
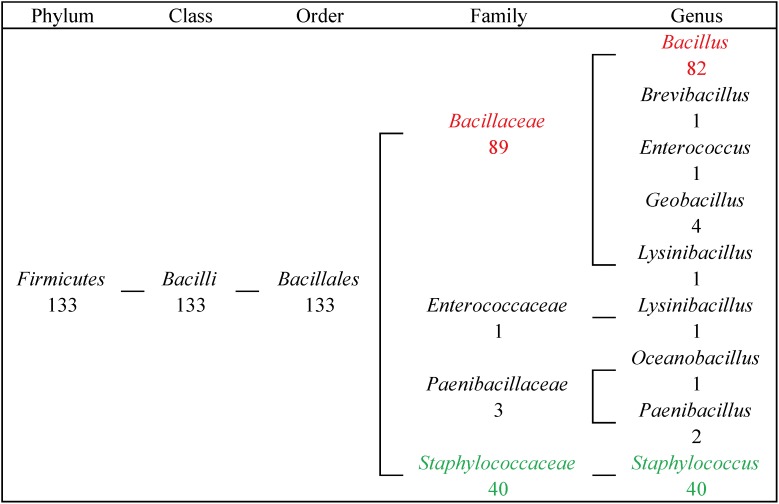
The taxonomic distribution of FosB in human gut microbiota. Numbers of homologues at different taxonomic levels were displayed. Red highlighted FosB from *Bacillus*; Green highlighted FosB from *Staphylococcus*.

From our results, 133 FosB homologues were distributed in four families of *Firmicutes*, and most of them were distributed in two genera. 89 FosB homologues were distributed in *Bacillaceae*, and 40 homologues were distributed in *Staphylococcaceae*. Similarly, 61.7% of *fosB*-containing bacteria belonged to *Bacillus*, and 30.0% belonged to *Staphylococcus* (Figure 2). These results indicated that *Bacillus* and *Staphylococcus* might play a dominant role in the resistance to fosfomycin.

### The Classification of FosB Based on the Sequence Similarity

**FIGURE 3:**
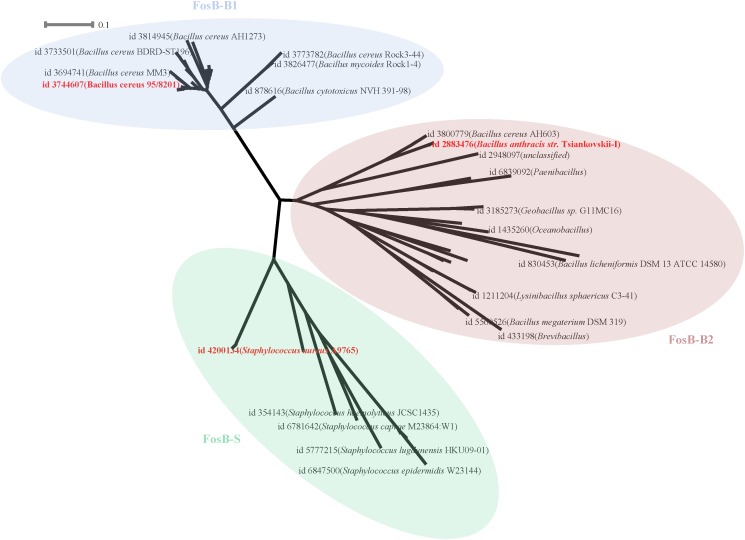
Phylogenetic tree of FosB. Different background colors of the three represented different clusters of FosB. Light blue, FosB-B1; Light red, FosB-B2; Light green, FosB-S. The representative sequences for subsequent determination of fosfomycin resistance were highlighted by the bold red font.

133 FosB homologues were divided into three clusters according to the phylogenetic tree, named as FosB-B1, FosB-B2, and FosB-S, respectively (Figure 3 and Supplementary Figure S1). The sequences of the same cluster had more similar characteristics, so we chose the representative sequence of each cluster to compare the resistant activity of the three clusters.

From the results of the sequence similarities of 14 FosB homologues with known 3D structures and all sequences of each cluster, the sequences with the highest similarity were selected for homology modeling (Supplementary Table S4). Finally, the selected sequence of FosB-B1 was distributed in *Bacillus cereus* 95/8201, FosB-B2 was distributed in *Bacillus anthracis str. Tsiankovskii-I* and FosB-S was distributed in *Staphylococcus aureus* A9765. These three representative FosB homologues were named as FosB-b1, FosB-b2, and FosB-s in the following calculations.

### Computational Sequence Analysis of FosB

**FIGURE 4:**
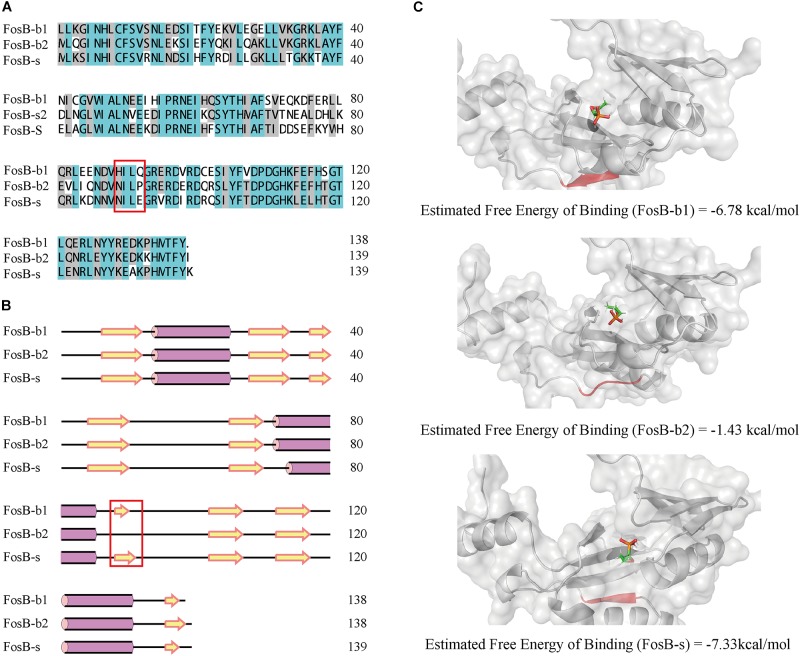
Comparative characterization of FosB-b1, FosB-b2, and FosB-s. **(A)** Multiple sequence alignment of FosB-b1, FosB-b2, and FosB-s. Different background colors indicated different sequences identity range. Malachite green, sequences identity = 100%; Gray, sequences identity > 50%; White, sequences identity < 50%. **(B)** The secondary structures prediction. The α-helix and β-strand were marked by the purple pillar and yellow arrow. **(C)** Molecular docking result structures of FosB-b1, FosB-b2 and FosB-s with fosfomycin. The residues and area of different β-sheet have been marked with red frame on sequences and secondary structures, and used red color to highlight them on the 3D structures.

By comparing primary sequences, the number of amino acids of FosB-b1, FosB-b2, and FosB-s was 138, 139, and 139, respectively. Also, FosB among them showed high similarity up to 75.54% (Figure 4A). Multiple sequence alignment of FosB revealed a highly conserved C-terminal and N-terminal regions.

From the secondary structures comparison of three FosB, it showed that FosB-b1, FosB-b2, and FosB-s all had three α-helixes, while the number and positions of β-sheets were different. There were nine β-sheets in FosB-b1 and FosB-s structures, but only eight in FosB-b2 (Figure 4B). Due to the special cyclic amino structure of Pro92 in FosB-b2, it was not easy to form β-sheet.

Furthermore, the molecular docking and MD simulations revealed the connections between fosfomycin and FosB. The free energy of the three FosB binding with fosfomycin were FosB-b2 (-1.43 kcal⋅mol^-1^) > FosB-b1 (-6.78 kcal⋅mol^-1^) > FosB-s (-7.33 kcal⋅mol^-1^) (Figure 4C and Supplementary Table S5). The unique β-sheets in FosB-s made the ligand of fosfomycin be bound to a non-polar cavity, while fosfomycin was more likely to bound to a polar environment in FosB-b1 and FosB-b2 (Figure 4B and Supplementary Figure S2). Thus, the lowest binding free energy was obtained from the complex formed by fosfomycin and FosB-s, which indicated the most stable structure of this complex and the highest activity of FosB-s from computational prediction.

### Resistance Evaluation of FosB to Fosfomycin

To analyse the resistant abilities of the three different FosB to fosfomycin, we formed the *fosB*-recombinant strains to express FosB-b1, FosB-b2, and FosB-s protein.

**FIGURE 5:**
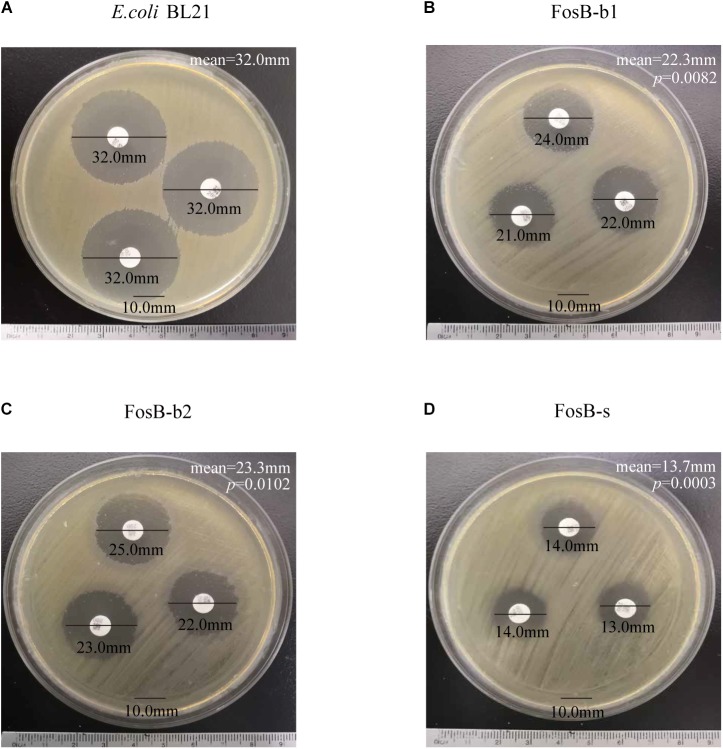
Kirby-Bauer susceptibility testing of **(A)** *E. coli* BL21, **(B)** FosB-b1, **(C)** FosB-b2 and **(D)** FosB-s. *p* values were calculated by student’s *T*-test, *p* < 0.05 was considered as statistically significant, versus the diameter of inhibition zone of *E. coli* BL21.

Kirby-Bauer susceptibility testing was used to demonstrate the fosfomycin resistance of the three recombinant strains, and *E. coli* BL21 was used as a control. The inhibition zones of the three *fosB*-recombinant *E. coli* was significant smaller than control strain (32.0 mm), and FosB-s (13.7 mm) < FosB-b1 (22.3 mm) < FosB-b2 (23.3 mm) (Figure 5). Thus, according to CLSI, the FosB-s belongs to I type (Intermediate, 13–15 mm), while FosB-b1 and FosB-b2 belong to S (Susceptible, >16 mm) though their inhibition zones are significant smaller than in control strain.

**FIGURE 6:**
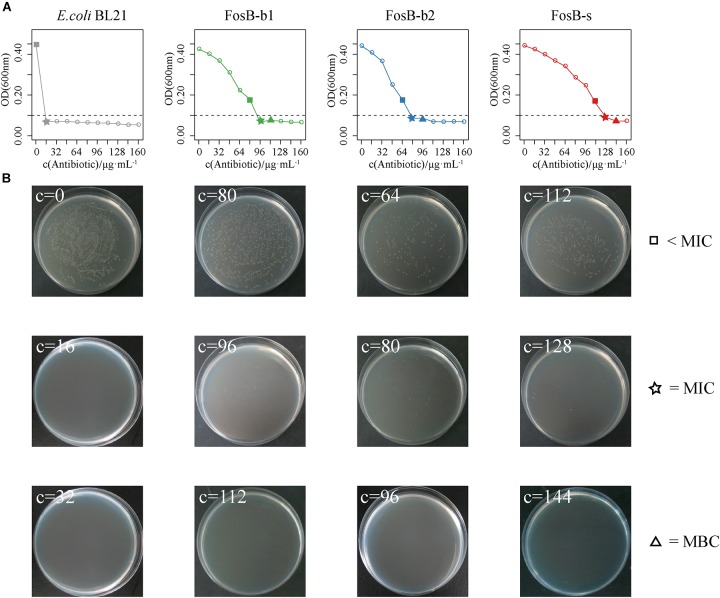
The analysis of fosfomycin resistance among three recombinant bacteria of FosB-b1, FosB-b2, and FosB-s. **(A)** The growth (OD_600_) of *E. coli* BL21 and three recombinant bacteria under different fosfomycin concentrations (μg⋅mL^-1^) within 12 h. **(B)** Distribution of *E. coli* BL21 and three recombinant bacteria on fosfomycin with different concentrations in culture dish at 24 h. The square, pentagram, and triangle represented the concentration below MIC, MIC, and MBC, respectively, which were consistent with a line chart.

To further compare the specific fosfomycin resistance of these three FosB, the OD_600_ of *fosB*-recombinant bacteria were measured at different concentrations of fosfomycin. We compared the results of fosfomycin resistance between the concentration of *fosB*-recombinant bacteria was 10^7^ cfu⋅mL^-1^ (Supplementary Figure S3) and 10^6^ cfu⋅mL^-1^ (Supplementary Figure S4), it showed that fosfomycin resistance were more stable when the concentration was 10^6^ cfu⋅mL^-1^. There was visible antibacterial activity on FosB-b1 and FosB-b2 when the fosfomycin concentration reached 96 μg⋅mL^-1^ and 80 μg⋅mL^-1^, respectively (Figure 6A). While the fosfomycin concentration increased to 128 μg⋅mL^-1^, the growth of FosB-s was inhibited, i.e., FosB-s had the highest resistant ability to fosfomycin. The MICs of fosfomycin on three recombinant bacteria were FosB-s (128 μg⋅mL^-1^) > FosB-b1 (96 μg⋅mL^-1^) > FosB-b2 (80 μg⋅mL^-1^) (Supplementary Table S6).

To obtain the MBCs of fosfomycin on the three FosB, their distributions for different concentrations of fosfomycin in culture dish at 37°C for 12 h were observed (Figure 6B). It was shown that no FosB-b1 colonies were found on the dish with 112 μg⋅mL^-1^ fosfomycin, no FosB-b2 colonies were found on the dish with 96 μg⋅mL^-1^ fosfomycin, and no FosB-s colonies were found on the dish with 144 μg⋅mL^-1^ fosfomycin, respectively. Together, the FosB-s showed the highest fosfomycin resistance with the MBCs results of FosB-s (144 μg⋅mL^-1^) > FosB-b1 (112 μg⋅mL^-1^) > FosB-b2 (96 μg⋅mL^-1^) (Supplementary Table S6). Furthermore, the accurate values of MICs and MBCs of fosfomycin on the three *fosB*-recombinant strains might help us in choosing more suitable fosfomycin-like antibiotics.

## Discussion

Several studies have reported that resistance variations are harmful to the environment and human health (Wright, 2010). In this study, we identified the distribution of all ARPs systematically in the HMP reference database firstly. From the results of ARDB, 5,808 ARPs’ sequences identified in the HMP reference database were obtained and distributed in 238 categories. However, from CARD, 29,045 ARPs’ sequences obtained, existed in 520 categories. The reason for the vast differences in the number of sequences may be the difference in the initial amounts of sequences in the corresponding databases (Supplementary Table S3).

From our results, FosB exhibited one of most homologues among all ARPs in human microbiota, which was consistent with the results from current studies (Thompson et al., 2014; Fu et al., 2016). On the other side, FosB was mainly identified in *Bacillus* and *Staphylococcus*. Therefore, it indicated that most of the bacteria in human microbiota might be sensitive to fosfomycin. However, how to control the horizontal gene transfer (HGT) of *fosB* from these two bacteria to others remains an central issue to fosfomycin resistance.

It was reported that FosB had been identified in *Staphylococcus* *aureus* and *Bacillus* *subtilis* (Cao et al., 2001; Thompson et al., 2014), which was consistent with our results, although we also identified FosB in other seven genera. Furthermore, we confirmed that the 133 FosB homologues could be classified into three clusters, and they exhibited distinct sequential, structural and functional differences. From the docking and dynamic analysis, we found FosB-b2 showed the highest free energy of binding due to the lack of β-sheet (Figure 4C). Biological validations also established that the fosfomycin-resistant activities of three groups of FosB homologues were FosB-S > FosB-B1 > FosB-B2. It is noteworthy that although the FosB-b1 and FosB-b2 belong to the susceptible type according to CLSI, they exhibit significant higher resistance activity than control strain. These results suggested that fosfomycin exhibited good bactericidal activity on different FosB clusters, and more effective strategies to control the proliferation of resistant organisms should be considered.

We also have investigated the prevalence of FosB in the gut microbiota of populations worldwide, based on public metagenome datasets (Qin et al., 2012; Yatsunenko et al., 2012; Chatelier et al., 2013; Karlsson et al., 2013; Lim et al., 2014; Feng et al., 2015; Zeller et al., 2015; Suguru et al., 2016). However, these results suggested that FosB was only distributed in the Korean community (Supplementary Table S7). The potential explanations for this result might be due to the following reasons. Firstly, the available public database we used originated from healthy individuals, i.e., people who took antibiotics in nine countries were excluded. Secondly, fosfomycin is mainly applied on poultry (Wang et al., 2017), and respiratory tract (Montgomery et al., 2014) of livestock at present, and studies on fosfomycin utilization for human are scarce. Finally, the bacteria containing *fosB* might tend to be existing in different genera in other parts of the body, like bone (Wittmann, 1980), lung (Adam and Ritscher, 1981).

Considering the increasing of fosfomycin application (Stock, 2015; Popovic et al., 2010) and homological sequences of FosB, therapy with fosfomycin alone led to the emergence of fosfomycin resistance quickly, and the potential threat of HGT (Xu et al., 2017) speed of resistance genes was getting faster. We can take advantage of the fact that different concentrations of fosfomycin were effective against various bacteria, particularly FosB-S cluster, given that it had the highest MBC to fosfomycin. Also, although *fosB* is rarely found in current public gut metagenomic databases, *fosB* in the environment can still be transferred into human organs. It is worthy to pay more attention to the molecular mechanisms of FosB resistance.

In summary, this work presented a macroscopic analysis of ARPs in ARDB and CARD and analyzed the taxonomic distribution of FosB. Our results indicated that although FosB had more homologues, it was mainly distributed in *Staphylococcus* and *Bacillus* genera. Moreover, they were divided into three clusters based on their phylogenetic relationship. Overall, the resistant activity comparisons showed that FosB from *Staphylococci* was more resistant to fosfomycin than from other bacteria, which might be a potential risk to become a pervasive resistance bacteria.

## Availability of Data and Material

All data generated in this manuscript are included in additional files.

## Author Contributions

JL conceived and designed the study. ZS, XW, XZ, SJ, YL, OA, LQ, PL, and JL collected data and performed the analyses. JL, ZS, and XW interpreted the data and wrote the manuscript. All authors read and approved the final manuscript.

## Conflict of Interest Statement

The authors declare that the research was conducted in the absence of any commercial or financial relationships that could be construed as a potential conflict of interest.

## Abbreviations

ARP: Antibiotic resistance protein
ARDB: Antibiotic Resistance Genes Database
BLASTP: Basic local alignment search tool
CARD: Comprehensive Antibiotic Resistance Database
CLSI: Clinical Laboratory Standards Institute
HGT: Horizontal gene transfer
HMP: Human Microbiome Project
MBC: Minimum bactericidal concentrations
MD: Molecular dynamics
MIC: Minimum inhibitory concentrations
ML: Maximum likelihood
MH: Mueller-Hinton
PDB: Protein data bank database
