# Supplementary material for: Taxonomic Distribution of FosB in Human-Microbiota and Activity Comparison of Fosfomycin Resistance
Source: Front Microbiol. 2019 Feb 13;10:200. doi: 10.3389/fmicb.2019.00200 (PMC6381061; doi:10.3389/fmicb.2019.00200)
Supplement: Supplementary file 2 [file Table_2.docx]

**Supplementary Materials**

**Taxonomic Distribution of FosB in Human-Microbiota and Activity Comparison of** **Fosfomycin Resistance**

Ziwei Song^1,2,3#^, Xue Wang^1,5#^, Xingchen Zhou^1,5^, Su Jiang^1^, Yuanyuan Li^1^, Owais Ahmad^1,2,3^, Lianwen Qi^3,4^, Ping Li^2,3*^, Jing Li^1,5*^.

^1^ School of Life Science and Technology, China Pharmaceutical University, Nanjing, China.

^2^ State Key Laboratory of Natural Medicines, China Pharmaceutical University, Nanjing, China.

^3^ School of Traditional Chinese Pharmacy, China Pharmaceutical University, Nanjing, China.

^4^ Clinical Metabolomics Center, China Pharmaceutical University, Nanjing, China.

^5^ Key Laboratory of Drug Quality Control and Pharmacovigilance, China Pharmaceutical University, Nanjing, China.

**Correspondence:**:

Jing Li ([lj_cpu@126.com](mailto:lj_cpu@126.com))

Ping Li (liping2004@126.com)

**Figure S1.** The detailed phylogenetic tree of 133 FosB homologues. Different colors of the tree represented three clusters. Light blue: FosB-B1; Light red: FosB-B2; Light green: FosB-S. Red bold font symbolized representative sequence of each cluster.

**Figure S2. (A)** Root-mean-square deviation (RMSD) of fosfomycin (Fos) binding with FosB-b1, **(B)** FosB-b2 and **(C)** FosB-s. **(D)** 2D diagram of the chemical interactions between fosfomycin (Fos) with FosB-b1, **e** FosB-b2 and **f** FosB-s. Data information: RMSD data showed the average change in ligand atomic displacement with respect to the initial binding mode during the course of MD simulation. The ligand interactions could reflect the chemical interaction between FosB and Fos.

**Figure S3.** The growth (OD_600_) of three *fosB*-recombinant bacteria and *E. coli* BL21 with different concentrations of fosfomycin (µg·mL^-1^) were detected per 2 hours over 12 hours, which was diluted about 10^2^ fold. The final concentration of bacteria solution was 10^7^ cfu·mL^-1^.

**Figure S4.** The growth (OD_600_) of three *fosB*-recombinant bacteria and *E. coli* BL21 with different concentrations of fosfomycin (µg·mL^-1^) were detected per 2 hours over 12 hours, which was diluted about 10^3^ fold. The final concentration of bacteria solution was 10^6^ cfu·mL^-1^.
