# Supplementary figures and images for: Taxonomic Distribution of FosB in Human-Microbiota and Activity Comparison of Fosfomycin Resistance
Source: Front Microbiol. 2019 Feb 13;10:200. doi: 10.3389/fmicb.2019.00200 (PMC6381061; doi:10.3389/fmicb.2019.00200)

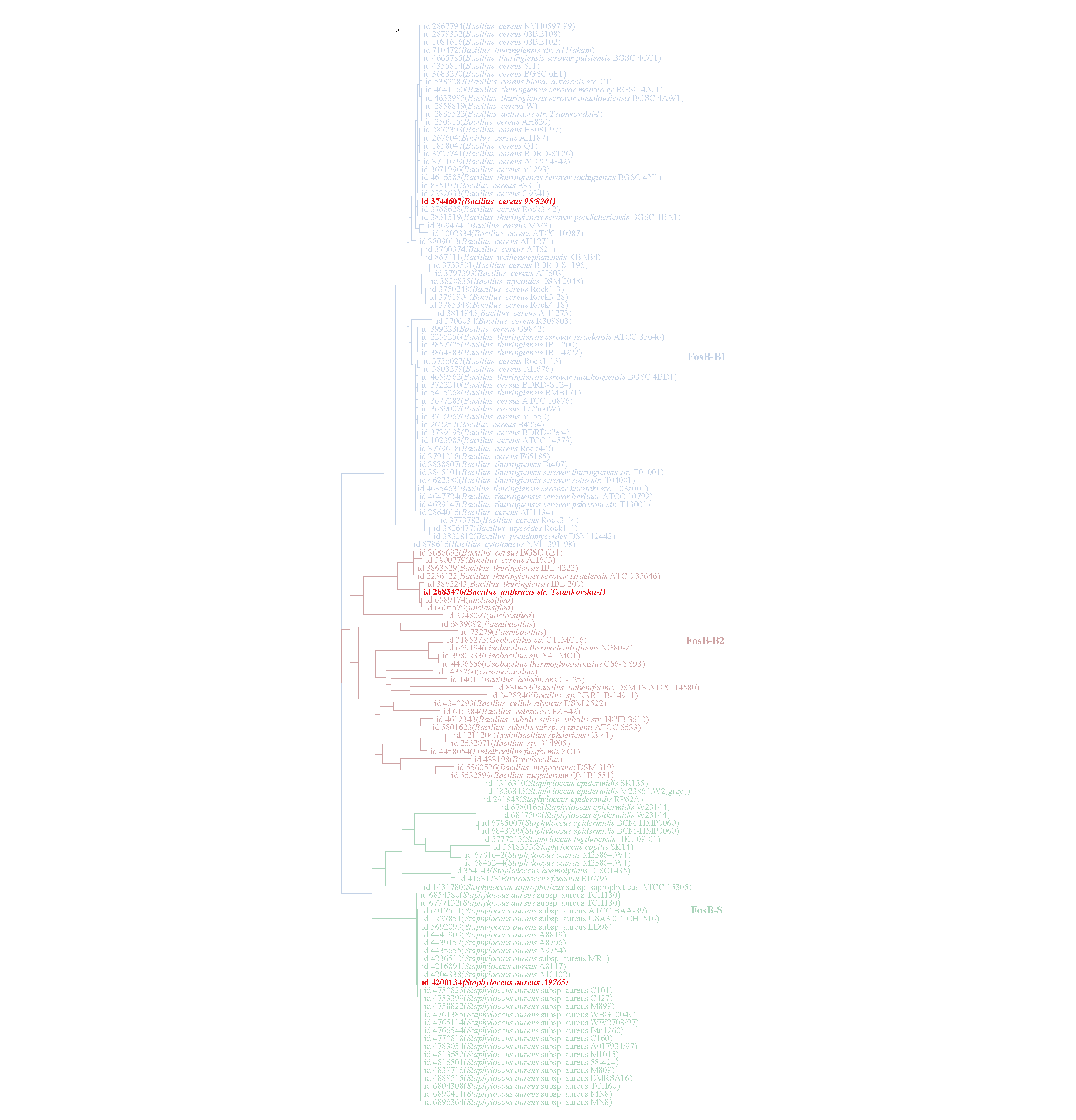

Supplement: Supplementary file 3 [file Image_1.TIF]

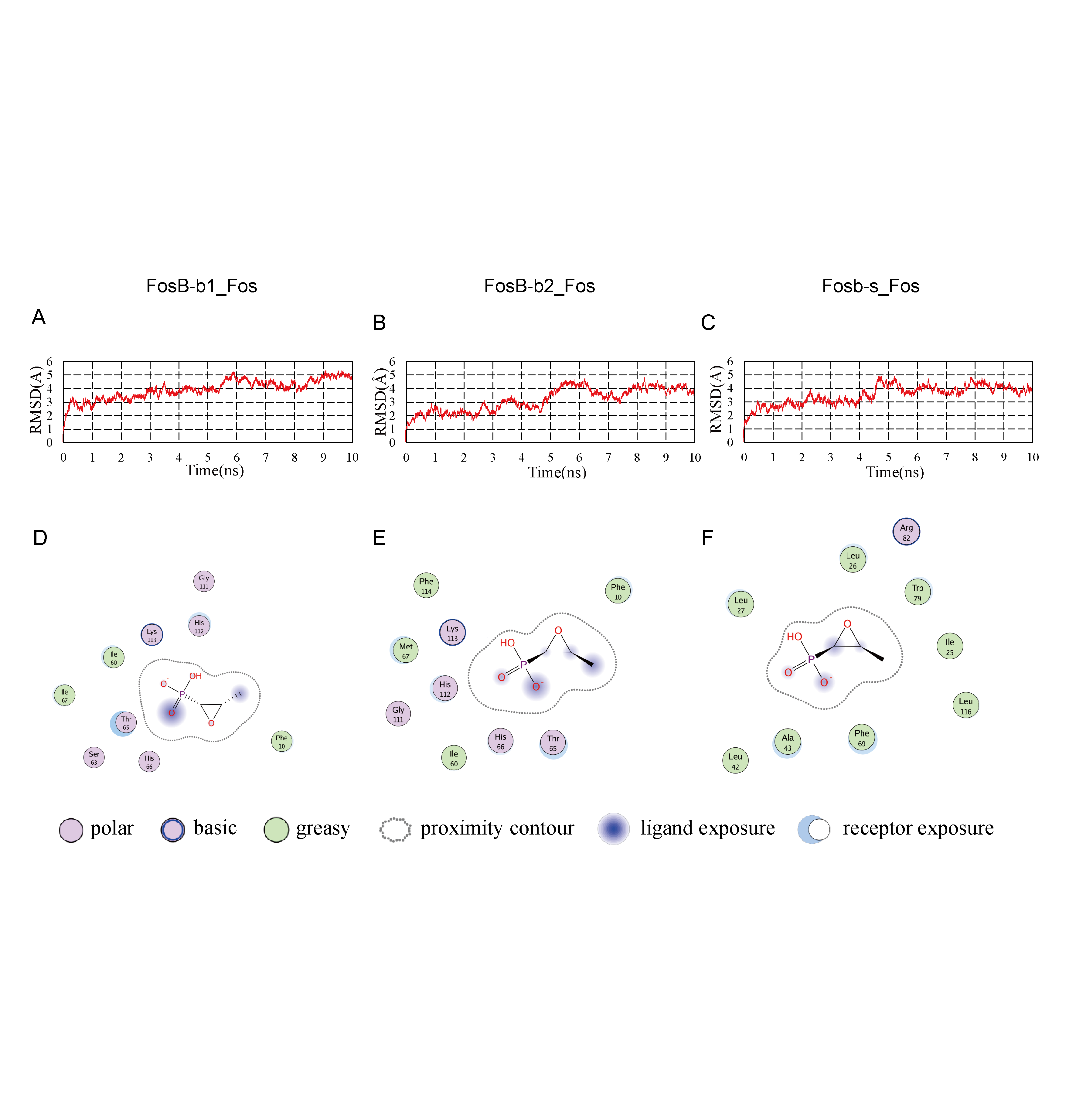

Supplement: Supplementary file 4 [file Image_2.TIF]

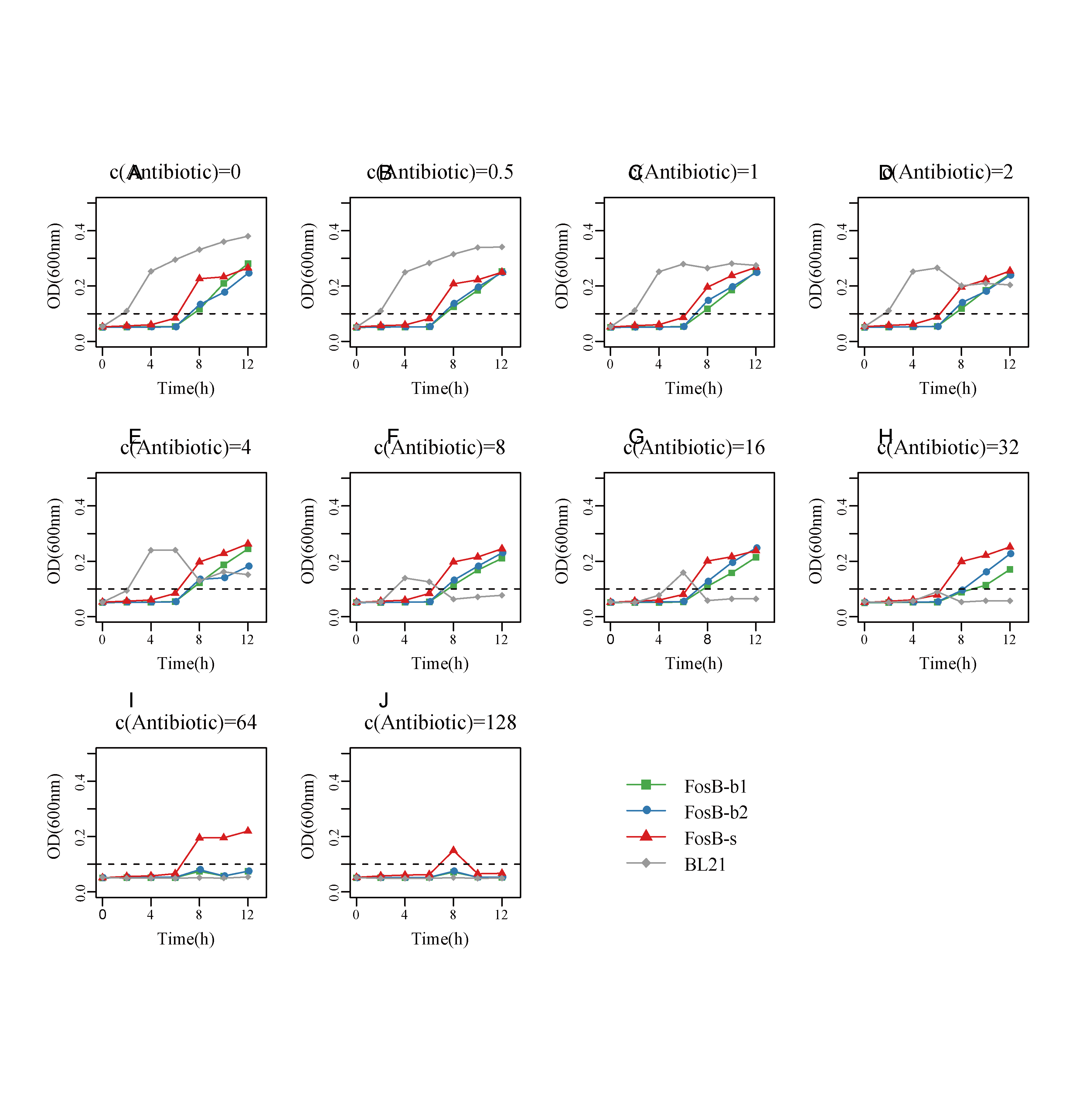

Supplement: Supplementary file 5 [file Image_3.TIF]

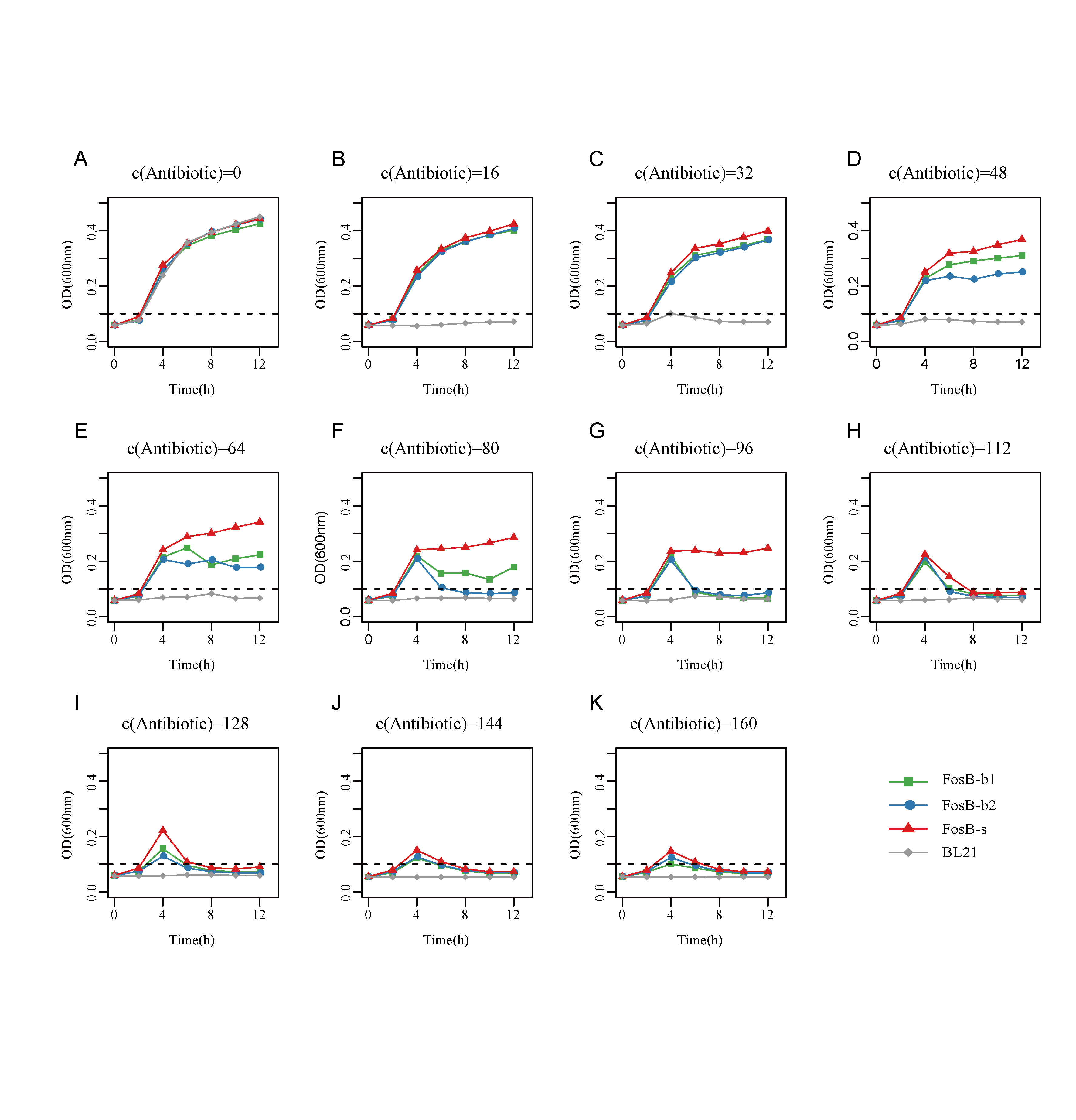

Supplement: Supplementary file 6 [file Image_4.TIF]
